# Orthogonal dual thiol–chloroacetyl and thiol–ene couplings for the sequential one-pot assembly of heteroglycoclusters

**DOI:** 10.3762/bjoc.10.160

**Published:** 2014-07-08

**Authors:** Michele Fiore, Gour Chand Daskhan, Baptiste Thomas, Olivier Renaudet

**Affiliations:** 1 Département de Chimie Moléculaire, UMR-CNRS 5250 & ICMG FR2607, Université Joseph Fourier, PB 53, 38041 Grenoble Cedex 9, France; 2 Institut Universitaire de France, 103 Boulevard Saint-Michel, 75005 Paris, France

**Keywords:** chemoselective ligation, heteroglycocluster, multivalency, multivalent glycosystems, one-pot synthesis

## Abstract

We describe the first one-pot orthogonal strategy to prepare well-defined cyclopeptide-based heteroglycoclusters (hGCs) from glycosyl thiols. Both thiol–chloroactetyl coupling (TCC) and thiol–ene coupling (TEC) have been used to decorate cyclopeptides regioselectively with diverse combination of sugars. We demonstrate that the reaction sequence starting with TCC can be performed one-pot whereas the reverse sequence requires a purification step after the TEC reaction. The versatility of this orthogonal strategy has been demonstrated through the synthesis of diverse hGCs displaying alternating binary combinations of α-D-Man or β-D-GlcNAc, thus providing rapid access to attractive heteroglycosylated platforms for diverse biological applications.

## Introduction

Multivalent carbohydrate–protein interactions are complex mechanisms that play key roles in biology [1]. To decipher, exploit or inhibit these recognition processes, a large variety of synthetic multivalent glycoconjugates have been developed over the last decade [2–4]. For a long time, these structures have capitalized on the utilization of a core scaffold decorated with identical sugars which are covalently linked through various spacers. While mimicking the multivalent sugar display of biological systems, these structures poorly reflect their inherent heterogenicity which hampers progresses towards the detailed elucidation of carbohydrate–protein interactions and the discovery of more selective ligands. Heteromultivalent ligands, namely heteroglycoclusters (hGCs), represent ideal structures to achieve this purpose [5]. A few recent reports described the construction of various hGCs based on the successive attachment of sugar residues on a core scaffold such as sugar [6–7], peptide [8–10], dendrimer [11–12], cyclodextrin [13–15] and polymer [16]. The most common synthetic strategy to build such hGCs relies on a fragment-coupling approach using thiol–ene coupling [17], copper(I)-catalyzed alkyne–azide cycloaddition (CuAAC) [18] or S_N_2 reaction [19]. In addition, orthogonal chemoselective ligations were proved more attractive strategies to prepare hGCs in high yields, in part because they require less synthetic and purification steps. For example, oxime and CuAAC ligations have been used in our group to prepare tetravalent structures displaying two sugars either in 2:2 or 3:1 relative proportions [20]. In the meanwhile, the group of A. Dondoni has developed a sequential orthogonal TEC in combination with CuAAC for grafting two different sugar motifs on calix[4]arene scaffold [21].

**Figure 1:**
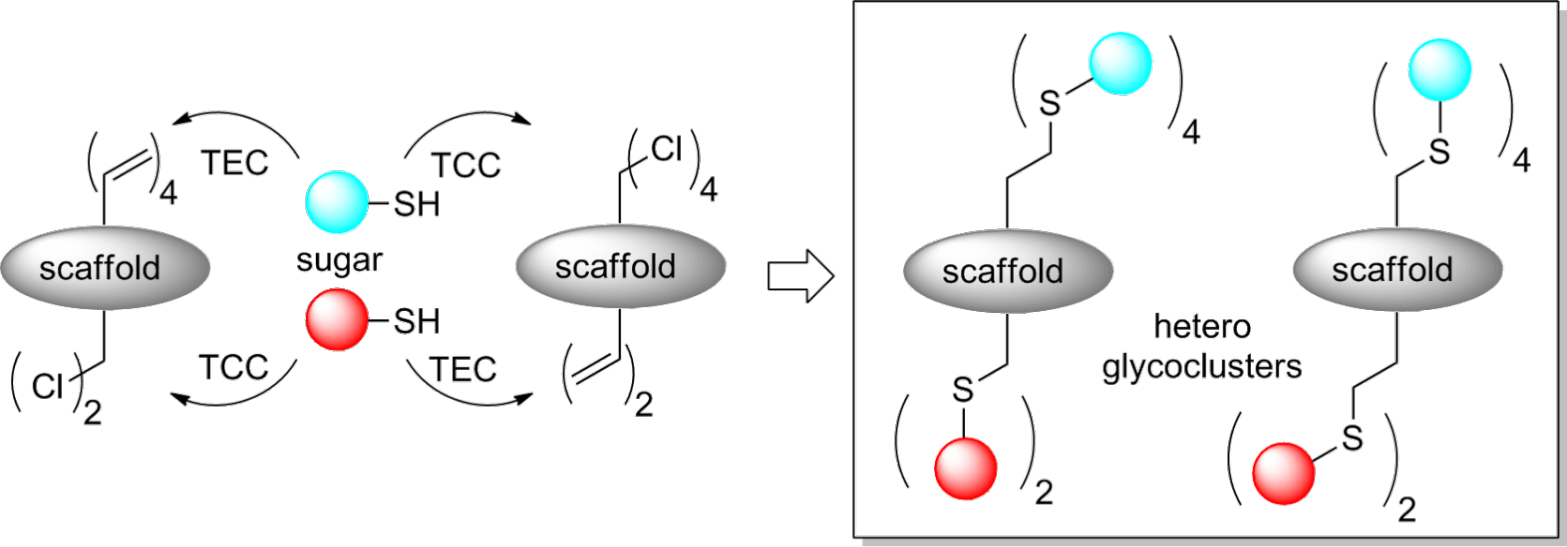
Chemical strategy for the construction of heteroglycoclusters.

Herein we report a new strategy based on both thiol–chloroactetyl coupling (TCC) and thiol–ene coupling (TEC) to prepare hGCs from glycosyl thiols and cyclopeptide scaffolds displaying chloroacetyl (ClAc) and allyloxycarbonyl (Alloc) groups and vice versa. We demonstrate that cyclopeptides regioselectively decorated with four sugars on one side, and two other sugars on the other side can be obtained either by a stepwise or a one-pot protocol depending on the reaction sequence (Figure 1). It should be mentioned that during the course of this study, the group of R. Roy has demonstrated the orthogonality of these two reactions for the growth of multifuncional dendrimers [22].

## Results and Discussion

**Scheme 1:**
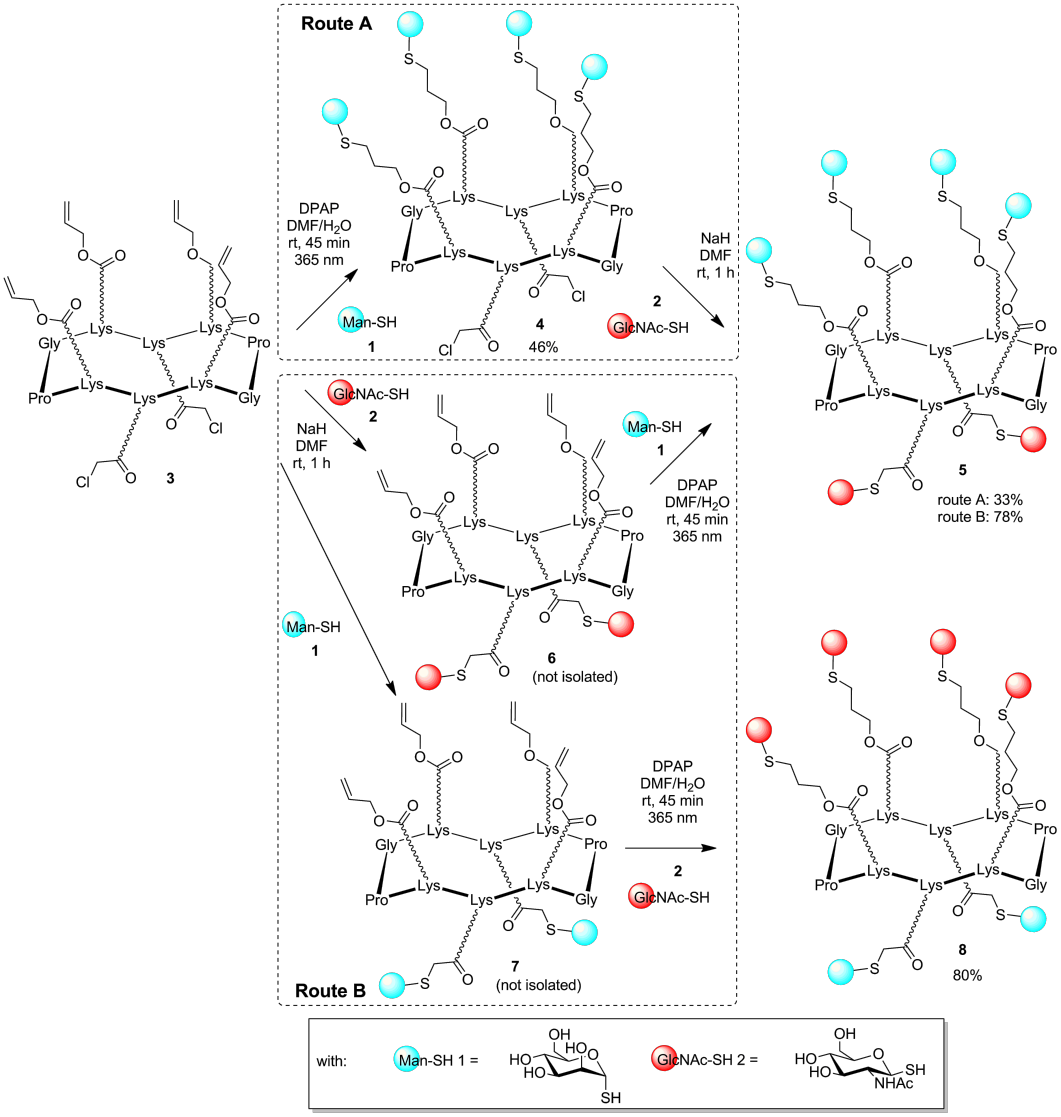
Stepwise (Route A) and sequential one-pot (Route B) synthesis of hGCs.

Owing to their straightforward access, their high nucleophilicity and the stability of thioether conjugates, glycosyl thiols [23–24], α-D-ManSH **1** and β-D-GlcNAcSH **2** have been selected for this study (Scheme 1). Such derivatives have proved to be useful in bioconjugates chemistry [25] and for the preparation of thioether-linked tetravalent glycocyclopeptides which have shown highest inhibition against a model lectin in comparison with analogues bearing oxime and triazole linkage [26]. Glycosyl thiols α-D-ManSH **1** and β-D-GlcNAcSH **2** were prepared from the corresponding bromo peracetyl and chloro peracetyl sugars by treatment with potassium thioacetate followed by de-*O*-acetylation under standard conditions [24].

Cyclopeptide **3** displaying two orthogonal functionalities, i.e., four lysine residues functionalized with Alloc groups [27] pointing on the upper face, and two lysine residues protected with chloroacetyl moiety at the lower face has been prepared. To evaluate the importance of the reaction sequences, we first performed the TEC reaction using α-D-ManSH **1**. This reaction was carried out in a mixture of DMF and H_2_O under UV irradiation (λ = 365 nm) in the presence of 2,2-dimethoxy-2-phenylacetophenone (DPAP) as a radical initiator (Scheme 1, route A). In previous studies [26], we observed that the TEC reaction requires the utilization of 3 equivalents of sugar per reaction site to be complete. Disappearance of starting material was indeed observed by reversed-phase HPLC after 45 minutes. The formation of the desired intermediate **4** having two chloroacetyl groups on the other side was confirmed by ESI mass spectrometry (see Supporting Information File 1 and Table 1). As expected, the chloroacetyl groups remained unreactive under these conditions as no partially glycosylated product was observed. Even though the HPLC profile of the crude mixture showed a clean reaction mixture, we were aware that the remaining presence of **1** could lead to the formation of an unwanted mixture of products. However we performed the next TCC reaction without further purification. The reaction occurred with a slight excess of **2** (1.2 equiv per reactive site) in the presence of NaH in dry DMF. Expectedly, this route gave a heterogeneous mixture of inseparable products, thus indicating that removal of the unreacted excess of sugar **1** is mandatory to avoid its addition during the TCC reaction. After purification, compound **4** was obtained in 46% yield and subsequently subjected to the TCC reaction with β-D-GlcNAcSH **2** under conditions described above. Compound **5**, wherein α-D-Man and β-D-GlcNAc occupied at the upper and the lower domains of the scaffold, respectively, was obtained after 1 hour as confirmed by HPLC and MS analyses (Table 1).

**Table 1:** Analytical data of the hGCs.

compound	yield (%)^a^	MS calc^b^	MS found^c^	*t*_R_ (min)^d^

**4**	46 (6.9 mg)	2351.9	2352.0	9.71
**5** (route A)	33 (2.6 mg)	2753.1	2753.2	8.24
**5** (route B)	78 (13.8 mg)	2753.1	2753.2	8.24
**8**	80 (10.2 mg)	2835.8	2836.0	8.35
**11**	77 (13.1 mg)	2666.1	2666.1	8.06
**13**	54 (14.4 mg)	2747.1	2747.2	8.06

^a^Yields were calculated on isolated compounds after HPLC purification. ^b^Calculated mass for [M + H]^+^. ^c^MS analysis was performed by electrospray ionization method in positive mode. ^d^RP-HPLC retention time using a linear gradient A–B, 95:5 to 0:100 in 20 min, flow: 1.0 mL/min, λ = 214 nm and 250 nm (column: nucleosil 300-5 C18; solvent A: 0.09% TFA in H_2_O, solvent B: 0.09% TFA in 90% acetonitrile).

We decided to investigate whether changing the reaction sequence could allow the one-pot assembly. We thus coupled β-D-GlcNAcSH **2** by TCC as the first step (Scheme 1, route B). Contrary to the previous route, we expected that the presence of unreacted sugar **2** (used in slight excess) might not interfere during the thiol–ene coupling as it should form disulfide adduct spontaneously. Therefore, the crude mixture was neutralized by addition of hydrochloric acid and compound **6** was used without further additional purification. α-D-ManSH **1** was then conjugated by TEC and compound **5** was obtained in 78% after purification. Interestingly no side product corresponding to the addition of **2** on the Alloc group was detected. We concluded that performing reactions in this order (route B) makes the one-pot assembly possible, faster and provides hGCs with higher yields (Table 1).

**Scheme 2:**
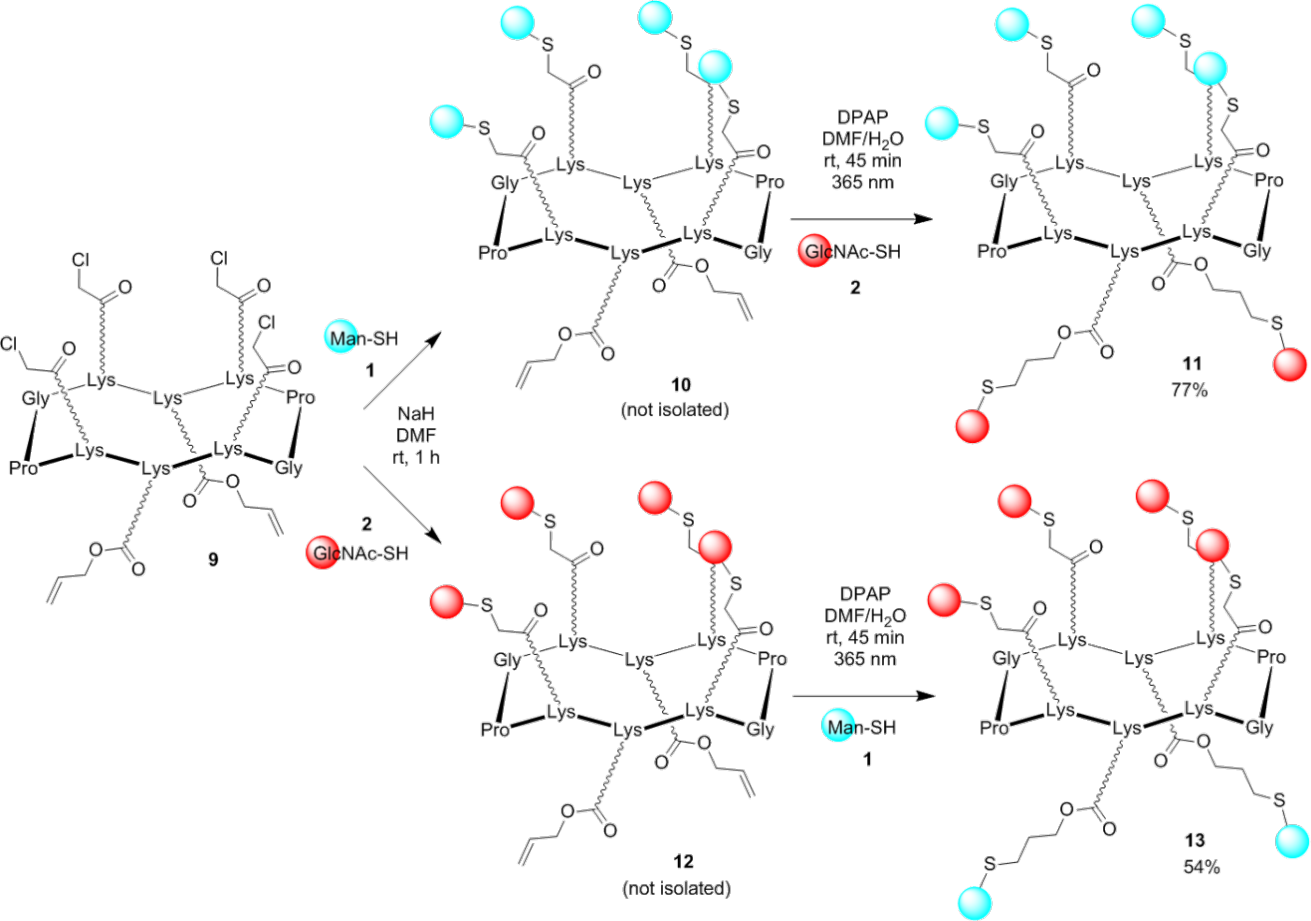
Synthesis of hGCs **11** and **13**.

To verify the efficiency and versatility of this protocol, we decided to perform similar sequence of reactions with cyclopeptide **9** having reactive functionalities in reverse orientation compared to **3**, i.e., four chloroacetyl and two Alloc moieties at the upper and the lower face, respectively (Scheme 2).

**Figure 2:**
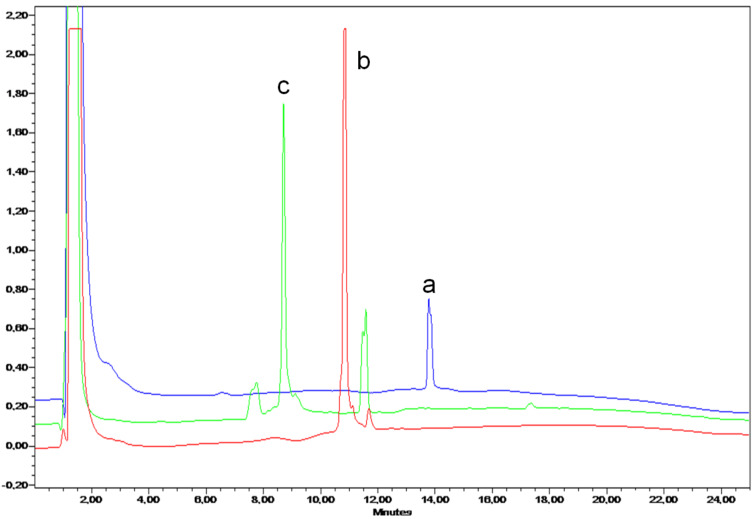
RP-HPLC profile of the one-pot synthesis of hGC **11** (linear A–B gradient: 5 to 100% B in 20 min, λ = 214 nm); (a, blue) cyclodecapeptide precursor **9**; (b, red) crude mixture of intermediate product **10**; (c, green) corresponds to crude mixture after TCC and TEC (**11**).

α-D-ManSH was used for the TCC reaction and β-D-GlcNAcSH for the subsequent TEC using a similar sequence of reactions described in Scheme 1. The HPLC profile of the crude mixture (Figure 2) showed that the successive TCC and TEC reactions give clean reaction mixtures to provide the hGC **11** with 77% yield.

The same strategy was followed to prepare compound **13** featuring two α-D-Man and four β-D-GlcNAc. No difference of reactivity was observed whatever the scaffold or the glycosyl thiol used. All these products were obtained in good yield after HPLC purification and gave the expected multicharged ions by electrospray mass spectrometry (Table 1).

## Conclusion

In summary, we have developed the first synthesis of hGCs using a one-pot orthogonal chemoselective route by using dual thiol–chloroacetyl and thiol–ene couplings. The effectiveness of this method was demonstrated through the coupling of multiple copies of α-D-ManSH and β-D-GlcNAcSH residues onto both addressable domains of cyclopeptide scaffolds displaying chloroacetyl and allyloxycarbonyl groups. While the first utilization of thiol–ene coupling in a stepwise approach requires an intermediate purification, a sequential one-pot assembly can be performed in good yields by starting with thiol–chloroacetyl coupling. This process is currently used in our laboratory for the construction of mutliantigenic synthetic vaccines against cancers.

## Experimental

**General details.** All chemical reagents were purchased from Aldrich (Saint Quentin Fallavier, France) or Acros (Noisy-Le-Grand, France) and were used without further purification. PyBOP was obtained from Calbiochem-Novabiochem (Merck Biosciences - VWR, Limonest, France). Analytical RP-HPLC was performed on a Waters system equipped with a Waters 600 controller and a Waters 2487 Dual Absorbance Detector. Analysis was carried out at 1.0 mL/min (EC 125/3 nucleosil 300-5 C18) with UV monitoring at 214 nm and 250 nm using a linear A–B gradient (buffer A: 0.09% CF_3_CO_2_H in water; buffer B: 0.09% CF_3_CO_2_H in 90% acetonitrile). Preparative separation was carried out at 22 mL/min (VP 250/21 nucleosil 300-7 C18) with UV monitoring at 214 nm and 250 nm using a linear A–B gradient (buffer A: 0.09% CF_3_CO_2_H in water; buffer B: 0.09% CF_3_CO_2_H in 90% acetonitrile). Mass spectrometry was performed using electrospray ionization on an Esquire 3000+ Bruker Daltonics in positive mode.

**General procedure for solid-phase peptide synthesis.** Assembly of all protected peptides was carried out on a synthesizer (Syro II, Biotage) using the Fmoc/*t*-Bu strategy and the Fmoc-Gly-Sasrin^TM^ resin. Coupling reactions were performed using, relative to the resin loading, 3 equiv of Fmoc-protected amino acid activated in situ with 3 equiv of PyBOP and 6 equiv of DIPEA in DMF (10 mL/g resin) for 30 min. Fmoc protecting groups were removed by treatment with a piperidine/DMF solution 1:4 (10 mL/g resin) for 10 min. Synthetic linear peptides were recovered directly upon acid cleavage (1% TFA in CH_2_Cl_2_). The resins were treated for 3 min repeatedly until the resin beads became dark purple. The combined washings were concentrated under reduced pressure, and white solid peptides were obtained by precipitation from diethyl ether.

**General procedure for peptide cyclization.** All linear peptides were dissolved in CH_2_Cl_2_ (0.5 mM) and the pH was adjusted to 8 by addition of DIPEA. PyBOP (1.2 equiv) was added and the solution was stirred at room temperature for 1 h. Evaporation of the solvent and precipitation in diethyl ether afforded the cyclic peptides as white solids.

**General procedure for Boc deprotection.** All cyclic peptides were dissolved in CH_2_Cl_2_ and then a solution at 40% of trifluoroacetic acid in CH_2_Cl_2_ with 2.5% of water as scavenger was added. The reaction was run until disappearance of stating material (1 h). Evaporation of the solvent and precipitation in diethyl ether afforded the cyclic peptides as white solids.

**Cyclopeptide 3**. To a solution of partial protected cyclopeptide [Lys(Aloc)-Lys-Lys(Aloc)-Pro-Gly-Lys(Aloc)-Lys-Lys(Aloc)-Pro-Gly] (200 mg, 0.142 mmol) in dry DMF (10 mL) was added chloroacetic anhydride (100 mg, 0.304 mmol) and pH adjusted to 8 by adding 100 μL of DIPEA. The brown solution was left stirring for 2 h. The solvent was then evaporated, the brown residue was dissolved in a minimum amount of CH_2_Cl_2_ and then precipitated in Et_2_O. The dark-yellow precipitate was purified by HPLC obtaining **3** (157 mg, 70%) as white foam. Analytical RP-HPLC: *t*_R_ = 16.64 min (gradient: 5 to 100% B in 20 min); ESIMS^+^ (*m*/*z*): [M + H]^+^ calcd for C_70_Cl_2_H_111_N_16_O_20_, 1567.7; found, 1567.7.

**Homoglycocluster 4.** Route A: Compounds **1** (14 mg, 0.076 mmol) and **3** (10 mg, 0.0064 mmol) were dissolved in dry DMF and DPAP (2.0 mg, 0.008 mmol) was added. The solution was irradiated at 365 nm for 45 min. Compound **4** (6.9 mg, 46%) was obtained as a white foam. Analytical RP-HPLC: *t*_R_ = 9.71 min (gradient: 5 to 100% B in 20 min); ESIMS^+^ (*m*/*z*): [M + H]^+^calcd for C_94_Cl_2_H_158_N_16_O_40_S_4_, 2351.9; found, 2352.0.

**Heteroglycocluster 5.** Route A: Compounds **4** (6.9 mg, 0.029 mmol) and **2** (1.7 mg, 0.0696 mmol) were dissolved in dry DMF (300 µL) and NaH (0.28 mg, 0.0696 mmol) was added. The heterogeneous solution was left stirring 2 h at rt. The crude mixture was then purified at HPLC obtaining **5** (2.6 mg, 33%) as a white foam. Analytical RP-HPLC: *t*_R_ = 8.24 min (gradient: 5 to 100% B in 20 min); ESIMS^+^ (*m*/*z*): [M + H]^+^ calcd for C_110_H_187_N_18_O_60_S_6_, 2753.1; found, 2753.2.

**Heteroglycocluster 5.** Route B (one-pot assembly). Compounds **2** (3.6 mg, 0.015 mmol) and **3** (10 mg, 0.0064 mmol) were dissolved in dry DMF (300 µL) and NaH (0.5 mg, 0.015 mmol) was added. After stirring 1 h at room temperature, analytical HPLC indicated complete disappearance of **3** and the appearance of a new product corresponding to compound **6**. Analytical HPLC: *t*_R_ = 11.34 (gradient: 5 to 100% B in 20 min); ESIMS^+^ (*m*/*z*): [M + H]^+^ calcd for C_86_H_139_N_18_O_30_S_2_, 1968.0; found, 1969.3. The crude mixture was treated with 1% HCl aqueous solution (150 µL) then compound **1** (14.52 mg, 0.0768 mmol) and DPAP (1.96 mg, 0.0077 mmol) were added. The solution was irradiated at 365 nm for 45 min. Heteroglycocluster **5** was obtained as a white foam after HPLC purification. Yield: 78%; (13.8 mg); analytical HPLC: *t*_R_ = 8.24 min (gradient: 5 to 100% B in 20 min); ESIMS^+^ (*m*/*z*): [M + H]^+^ calcd for C_110_H_187_N_18_O_60_S_6_, 2753.1; found, 2753.2.

**Heteroglycocluster 8**. Heteroglycocluster **8** wad obtained from **1** (2.4 mg, 0.0122 mmol), **3** (8 mg, 0.0051 mmol) and **2** (14.2 mg, 0.06 mmol) as described for **5**. Yield: 80% (10.2 mg); analytical RP-HPLC: *t*_R_ = 8.35 min (gradient: 5 to 100% B in 20 min); ESIMS^+^ (*m*/*z*): [M + H]^+^ calcd for C_114_H_193_N_20_O_50_S_6_, 2835.8; found, 2836.0.

**Cyclopeptide 9**. To a solution of the partial protected cyclopeptide [Lys-Lys(Aloc)-Lys-Pro-Gly-Lys-Lys(Aloc)-Lys-Pro-Gly] (871 mg, 0.70 mmol) in dry DMF (40 mL) was added chloroacetic anhydride (601.2 mg, 3.36 mmol) and the pH was adjusted to 8 by adding 250 μL of DIPEA. The brown solution was left stirring for 4 h. Solvent was then evaporated; the brown residue was dissolved in a minimum amount of CH_2_Cl_2_ and then precipitated in Et_2_O. The dark-brown precipitate was purified by HPLC obtaining **9** (459 mg, 42%) as white foam. Analytical RP-HPLC: *t*_R_ = 12.67 min (gradient: 5 to 100% B in 20 min); ESIMS^+^ (*m*/*z*): [M + H]^+^ calcd for C_66_Cl_4_H_105_N_16_O_18_, 1582.7; found, 1582.0.

**Heteroglycocluster 11.** Heteroglycocluster **11** was obtained from **9** (13 mg, 0.0084 mmol), **1** (8 mg, 0.0402 mmol) and **2** (11.9 mg, 0.0504 mmol) as described for **5**. Yield: 77% (13.1 mg); analytical RP-HPLC: *t*_R_ = 8.06 min (gradient: 5 to 100% B in 20 min); ESIMS^+^ (*m*/*z*): [M + H]^+^ calcd for C_106_H_180_N_18_O_48_S_6_, 2666.1; found, 2666.1.

**Heteroglycocluster 13**. Heteroglycocluster **13** was obtained from **9** (15 mg, 0.0097 mmol), **2** (11.0 mg, 0.0466 mmol) and **1** (11.4 mg, 0.058 mmol) as described for **5**. Yield: 54% (14.4 mg); analytical RP-HPLC: *t*_R_ = 8.06 min (gradient: 5 to 100% B in 20 min); ESIMS^+^ (*m*/*z*): [M + H]^+^ calcd for C_110_H_184_N_20_O_48_S_6_, 2747.1; found, 2747.2.

## Supporting Information

File 1 HPLC chromatograms and mass spectra of all compounds.

## References

[1] Varki A, Cummings R D, Esko J D (2009). Essentials of Glycobiology.

[2] Kiessling L L, Gestwicki J E, Strong L E (2006). Angew Chem, Int Ed.

[3] Chabre Y M, Roy R (2010). Adv Carbohydr Chem Biochem.

[4] Renaudet O, Roy R (2013). Chem Soc Rev.

[5] Jiménez Blanco J L, Ortiz Mellet C, Garcia Fernández J M (2013). Chem Soc Rev.

[6] Patel A, Lindhorst T K (2002). Eur J Org Chem.

[7] Ortega-Muñoz M, Perez-Balderas F, Morales-Sanfrutos J, Hernandez-Mateo F, Isac-García J, Santoyo-Gonzalez F (2009). Eur J Org Chem.

[8] Katajisto J, Karskela T, Heinonen P, Lönnberg H (2002). J Org Chem.

[9] Lindhorst T K, Bruegge K, Fuchs A, Sperling O (2010). Beilstein J Org Chem.

[10] Keding S J, Danishefsky S J (2004). Proc Natl Acad Sci U S A.

[11] Deguise I, Lagnoux D, Roy R (2007). New J Chem.

[12] Wolfenden M L, Cloninger M J (2006). Bioconjugate Chem.

[13] Gómez-García M, Benito J M, Rodríguez-Lucena D, Yu J-X, Chmurski K, Ortiz Mellet C, Gutiérrez Gallego R, Maestre A, Defaye J, Garcìa Fernàndez J M (2005). J Am Chem Soc.

[14] Gómez-García M, Benito J M, Gutiérrez-Gallego R, Maestre A, Ortiz Mellet C, García Fernández J M, Jiménez Blanco J L (2010). Org Biomol Chem.

[15] Gómez-Garcia M, Benito J M, Butera A P, Ortiz-Mellet C, Garcia Fernàndez J M, Jiménez Blanco J L (2012). J Org Chem.

[16] Geng J, Mantovani G, Tao L, Nicolas J, Chen G, Wallis R, Mitchell D A, Johnson B R G, Evans S D, Haddleton D M (2007). J Am Chem Soc.

[17] Dondoni A (2008). Angew Chem, Int Ed.

[18] Rostovtsev V V, Green L G, Fokin V V, Sharpless K B (2002). Angew Chem, Int Ed.

[19] Elsner K, Boysen M M K, Lindhorst T K (2007). Carbohydr Res.

[20] Thomas B, Fiore M, Bossu I, Dumy P, Renaudet O (2012). Beilstein J Org Chem.

[21] Fiore M, Chambery A, Marra A, Dondoni A (2009). Org Biomol Chem.

[22] Kottari N, Chabre Y M, Shiao T C, Roy R (2014). Chem Commun.

[23] MacDougall J M, Zhang X-D, Polgar W E, Kharoyan T V, Toll L, Cashman J R (2004). J Med Chem.

[24] Bernardes G J L, Gamblin D P, Davis B G (2006). Angew Chem, Int Ed.

[25] Gingras M, Chabre Y M, Roy M, Roy R (2013). Chem Soc Rev.

[26] Fiore M, Berthet N, Marra A, Gillon E, Dumy P, Dondoni A, Imberty A, Renaudet O (2013). Org Biomol Chem.

[27] Eggimann G A, Buschor S, Darbre T, Reymond J-L (2013). Org Biomol Chem.

