# Supplementary material for: Orthogonal dual thiol–chloroacetyl and thiol–ene couplings for the sequential one-pot assembly of heteroglycoclusters
Source: Beilstein J Org Chem. 2014 Jul 8;10:1557–63. doi: 10.3762/bjoc.10.160 (PMC4142873; doi:10.3762/bjoc.10.160)
Supplement: File 1 — HPLC chromatograms and mass spectra of all compounds. [file Beilstein_J_Org_Chem-10-1557-s001.pdf]

Supporting Information

for

Orthogonal dual thiol–chloroacetyl and thiol–ene couplings for the sequential one-pot assembly of heteroglycoclusters

Michele Fiore[1], Gour Chand Daskhan[1], Baptiste Thomas[1] and Olivier Renaudet*[1,2]

[1]Département de Chimie Moléculaire, UMR-CNRS 5250 & ICMG FR2607, Université Joseph Fourier, PB 53, 38041 Grenoble Cedex 9, France and [2]Institut Universitaire de France, 103 Boulevard Saint-Michel, 75005 Paris, France.

E-mail: Olivier Renaudet***** - olivier.renaudet@ujf-grenoble.fr

*Corresponding author

HPLC chromatograms and mass spectra of all compounds

Compound 3.

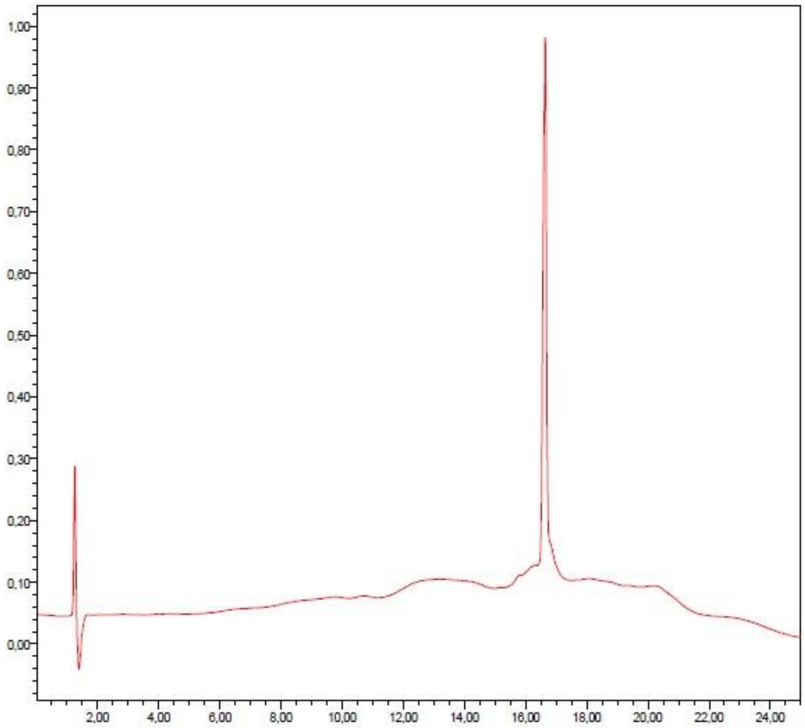

Analytical RP-HPLC: t_R = 16.64 min (gradient: 5 to 100% B in 20 min, λ = 214 nm).

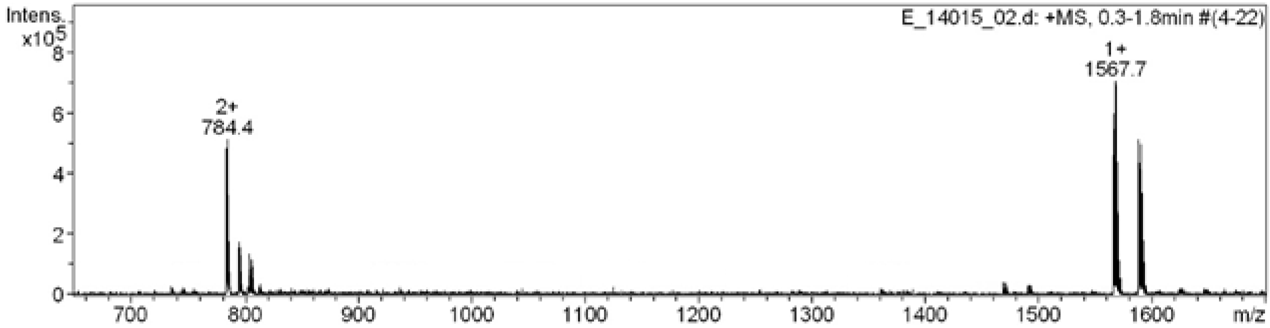

ESIMS$^+$ (m/z): $[M + H]^+$ calcd for $C_{70}H_{111}Cl_2N_{16}O_{20}$, 1567.7; found, 1567.7.

Compound 4.

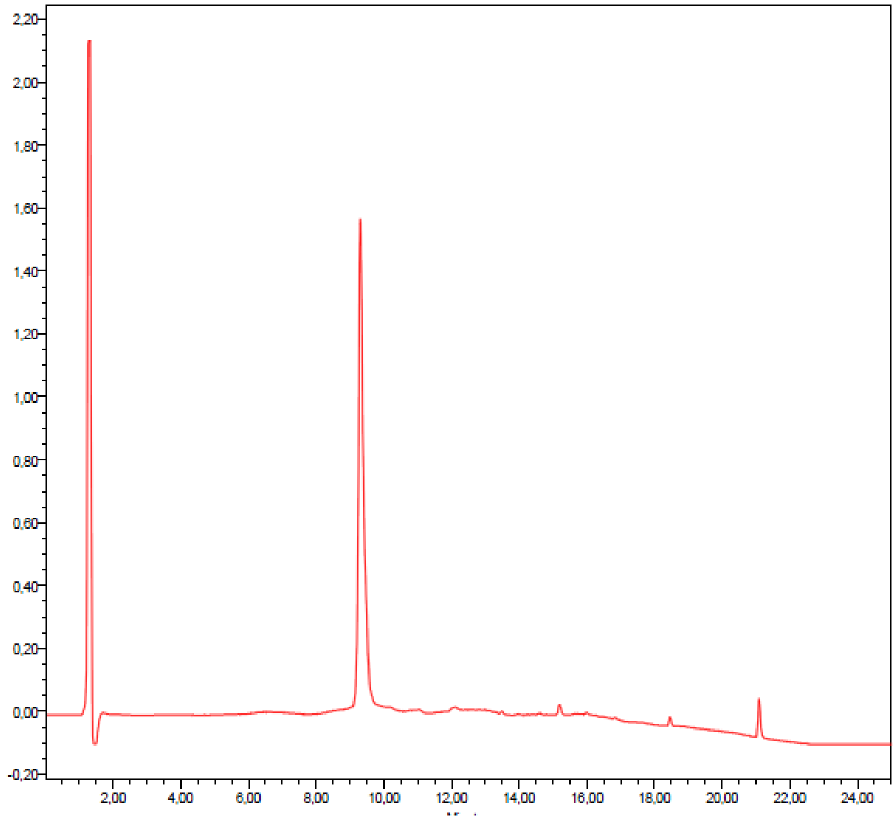

Analytical RP-HPLC: t_R = 9.71 (gradient: 5 to 100% B in 20 min, λ = 214 nm).

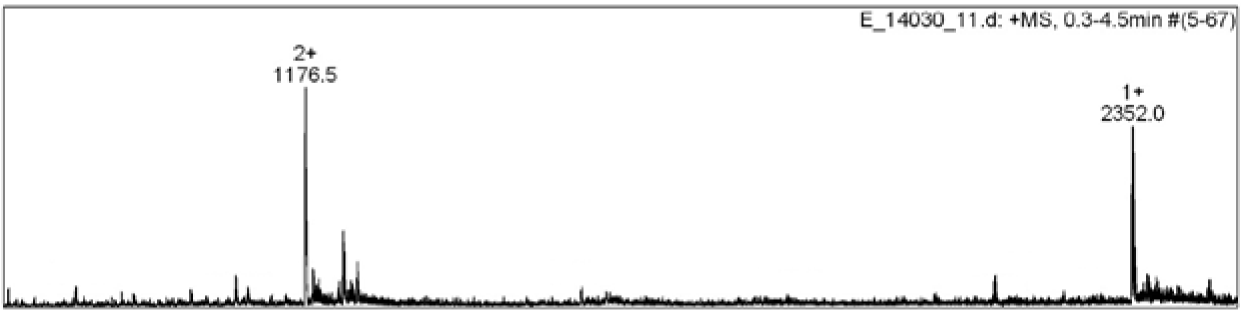

ESIMS$^+$ (m/z): $[M + H]^+$ calcd. for $C_{94}Cl_2H_{159}N_{16}O_{40}S_4$, 2351.9; found, 2352.0.

Compound 5.

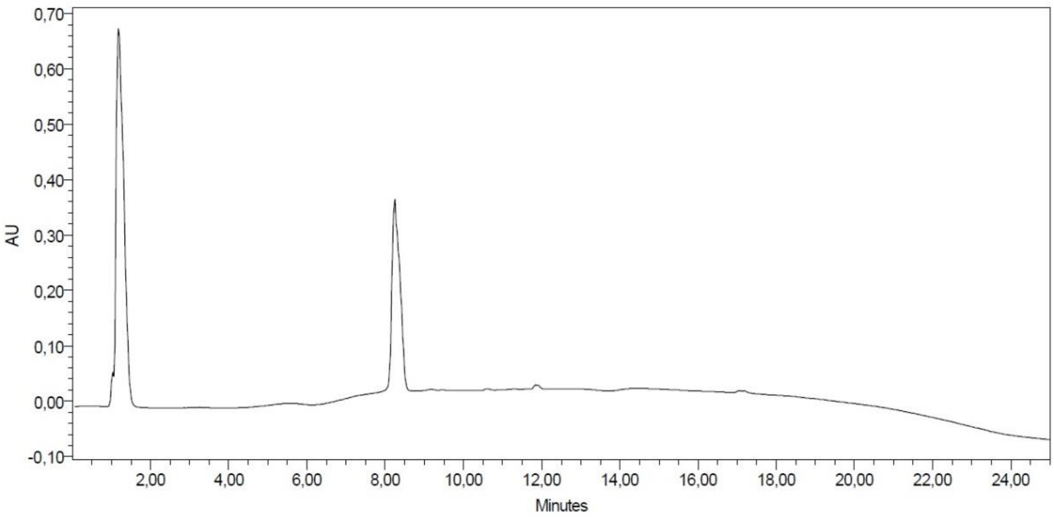

RP-HPLC: t_R = 8.24 (gradient: 5 to 100% B in 20 min, λ = 214 nm).

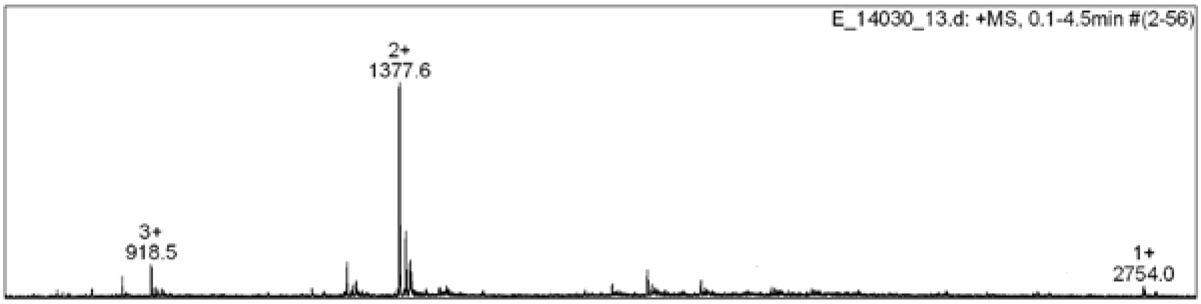

ESIMS$^+$ (m/z): [M + H]$^+$ calcd for $C_{110}H_{187}N_{18}O_{50}S_6$, 2753.1; found, 2753.2.

Compound 8.

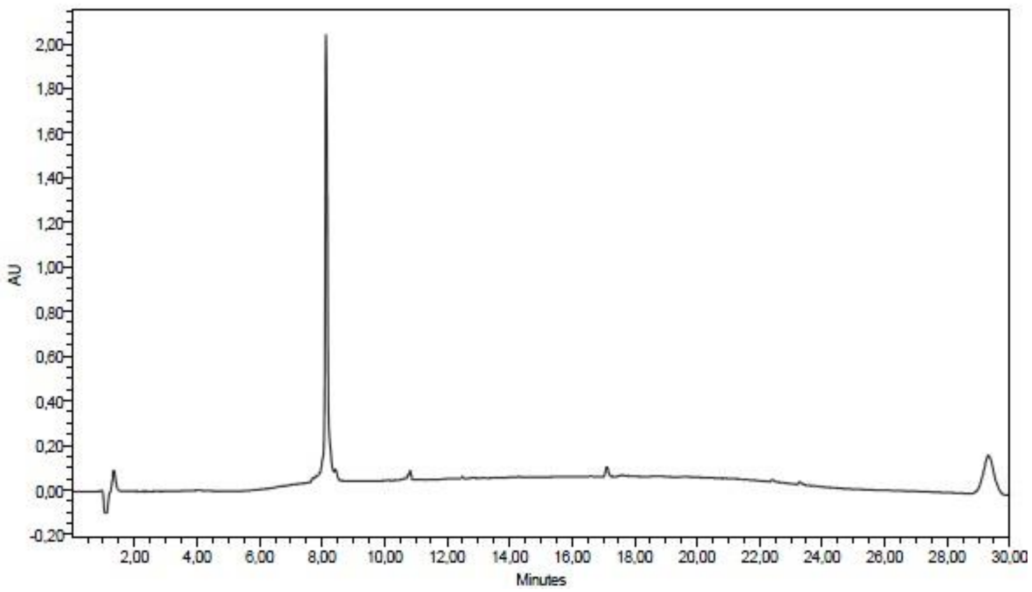

Analytical RP-HPLC: t_R = 8.35 min (gradient: 5 to 100% B in 20 min, , λ = 214 nm).

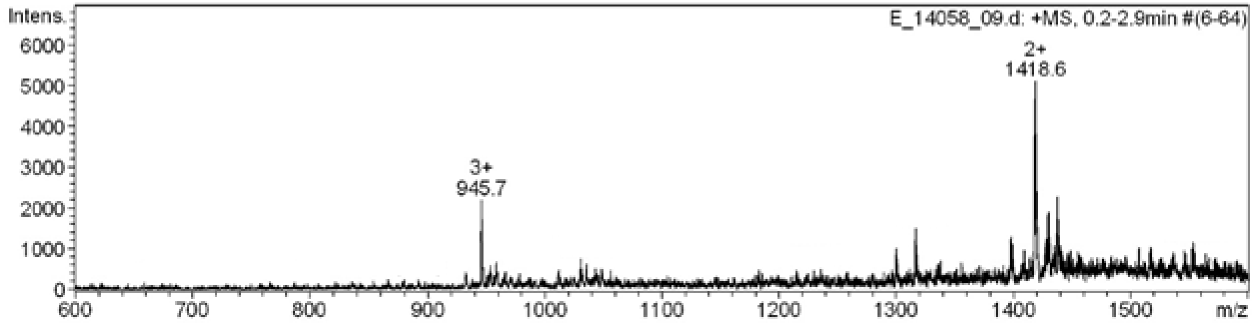

ESIMS$^+$ (m/z): $[M + H]^+$ calcd for $C_{114}H_{193}N_{20}O_{50}S_6$, 2835.8; found, 2836.0.

Compound 9.

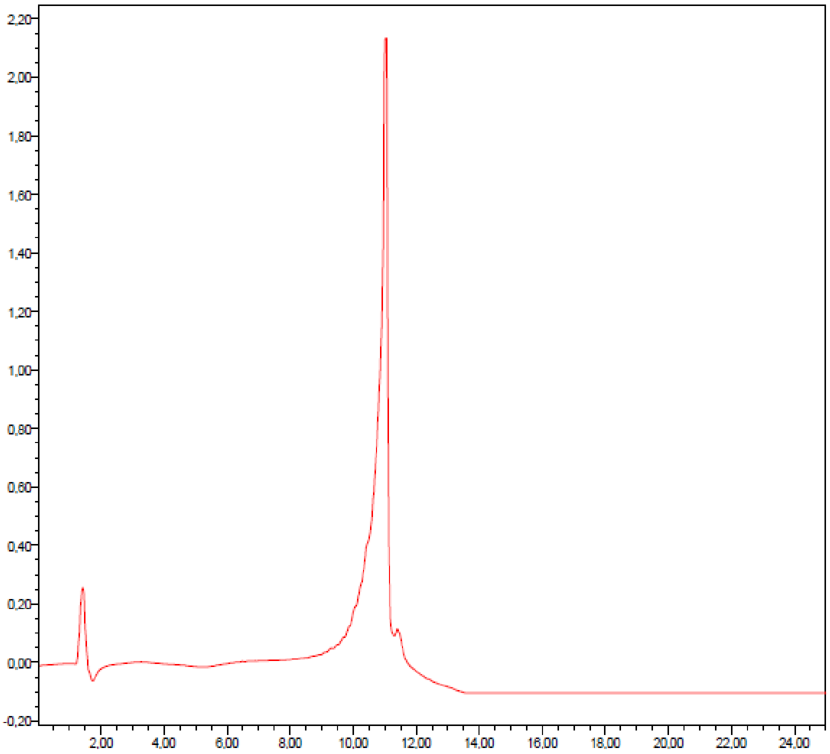

Analytical RP-HPLC: t_R = 11.67 min (gradient: 5 to 100% B in 20 min, , λ = 214 nm).

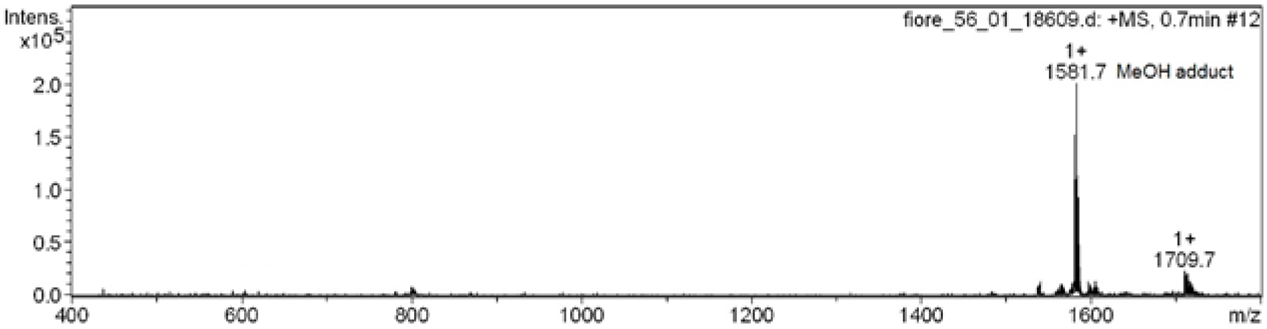

ESIMS$^+$ (m/z): $[M + MeOH]^+$ calcd for $C_{66}H_{105}Cl_4N_{16}O_{18}$, 1582.7; found, 1582.0.

Compound 11.

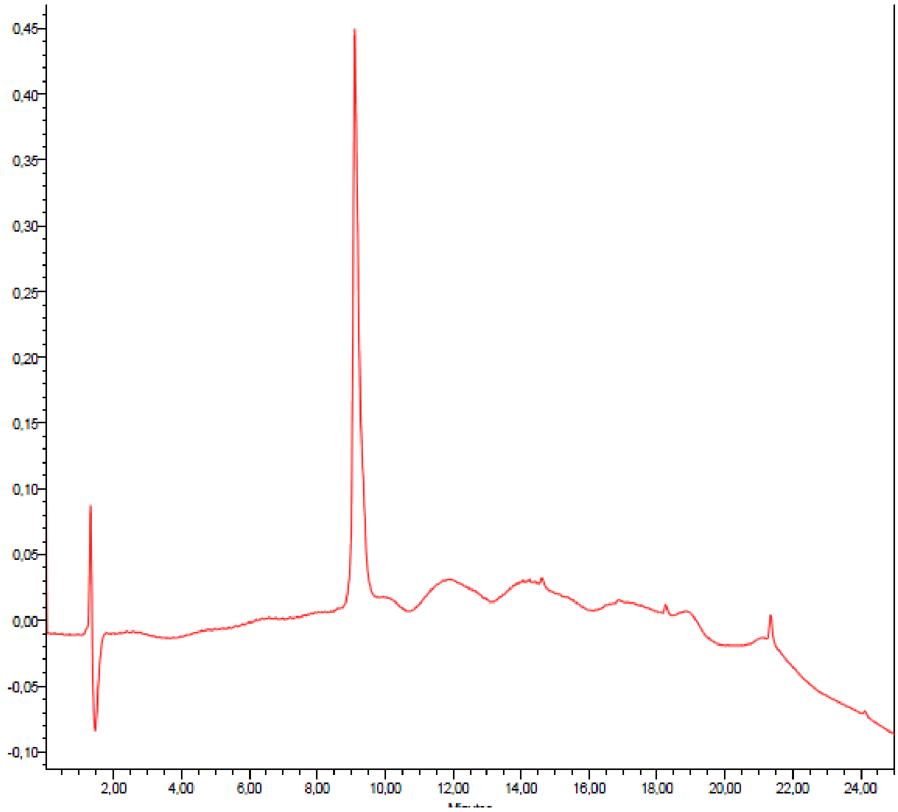

Analytical RP-HPLC: t_R = 8.06 min (gradient: 5 to 100% B in 20 min, , λ = 214 nm).

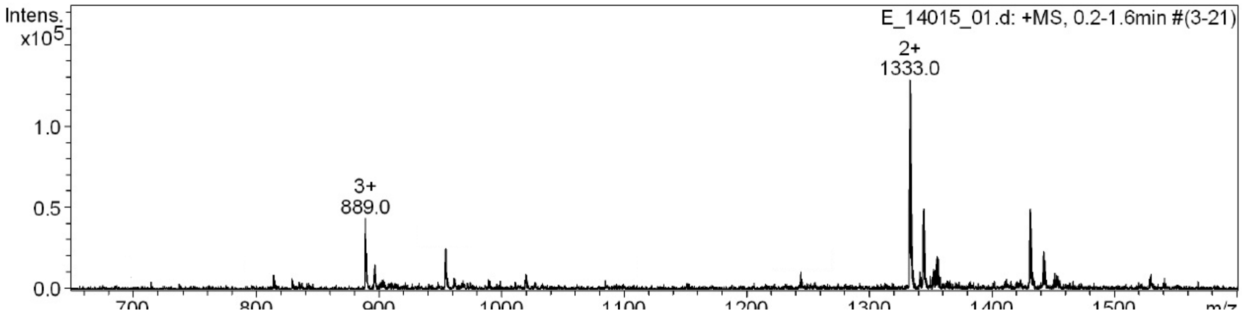

ESIMS$^+$ (*m*/*z*): $[M + H]^+$ calcd for $C_{106}H_{180}N_{18}O_{48}S_6$, 2666.1; found, 2666.1.

Compound 13.

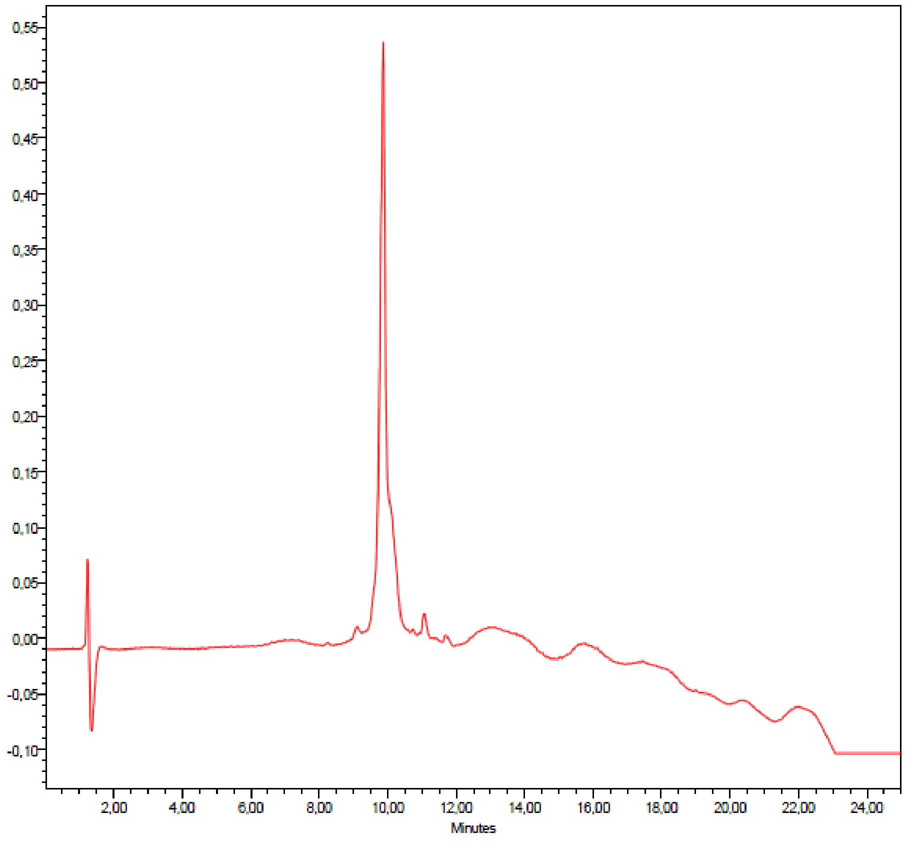

Analytical RP-HPLC: t_R = 10.06 min (gradient: 5 to 100% B in 20 min, λ = 214 nm).

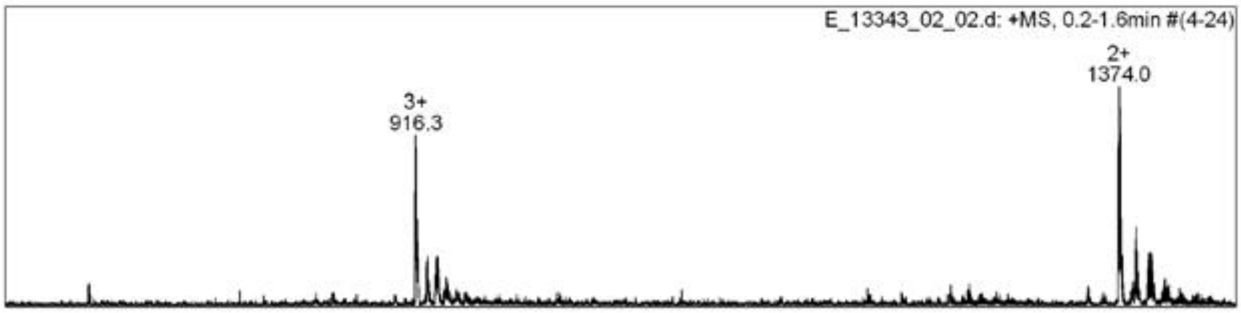

ESIMS$^+$ (m/z): $[M + H]^+$ calcd for $C_{110}H_{184}N_{20}O_{48}S_6$, 2747.1; found, 2747.2.
